# Aluminum particles generated during millisecond electric pulse application enhance adenovirus-mediated gene transfer in L929 cells

**DOI:** 10.1038/s41598-021-96781-y

**Published:** 2021-09-06

**Authors:** Angela Tesse, Franck M. André, Thierry Ragot

**Affiliations:** 1 CNRS, Institut Gustave Roussy, Université Paris-Saclay, Aspects métaboliques et systémiques de l’oncogenèse pour de nouvelles approches thérapeutiques, UMR 9018, 114 rue Edouard Vaillant, F-94805 Villejuif, France; 2 Université de Nantes, CNRS, INSERM, l’institut du thorax, 8 quai Moncousu, F-44000 Nantes, France

**Keywords:** Genetic vectors, Adenovirus, Nanoscale materials, Transfection, Permeation and transport, Electrical and electronic engineering

## Abstract

Gene electrotransfer is an attractive method of non-viral gene delivery. However, the mechanism of DNA penetration across the plasma membrane is widely discussed. To explore this process for even larger structures, like viruses, we applied various combinations of short/long and high/low-amplitude electric pulses to L929 cells, mixed with a human adenovirus vector expressing GFP. We observed a transgene expression increase, both in the number of GFP-converted cells and GFP levels, when we added a low-voltage/millisecond-pulse treatment to the adenovirus/cell mixture. This increase, reflecting enhanced virus penetration, was proportional to the applied electric field amplitude and pulse number, but was not associated with membrane permeabilization, nor to direct cell modifications. We demonstrated that this effect is mainly due to adenovirus particle interactions with aggregated aluminum particles released from energized electrodes. Indeed, after centrifugation of the pulsed viral suspension and later on addition to cells, the activity was found mainly associated with the aluminum aggregates concentrated in the lower fraction and was proportional to generated quantities. Overall, this work focused on the use of electrotransfer to facilitate the adenovirus entry into cell, demonstrating that modifications of the penetrating agent can be more important than modifications of the target cell for transfer efficacy.

## Introduction

Delivery of macromolecules or nano-objects with bioactive properties into live cells either grown in vitro or constitutive of living tissues is a fundamental and irreplaceable procedure in many fields of biotechnology and more and more in various fields of therapeutics. Electrotransfer is one major tool for this aim. It intends to use external electric pulses to induce or facilitate the penetration of bioactive agents into target cells. So far, electrotransfer has been mainly used for intracellular delivery of plasmid DNA in a large range of cells and tissues. Moreover, diversification of pulse types and their combination, associated to a variety of devices for pulse delivery, have allowed to extend the scope of gene electrotransfer applications^1,2^. Electrogenetherapy strategies like DNA vaccination and/or cytokine-based immunotherapy to stimulate anti-tumor immunity are promising strategies to fight cancer^3^, as suggested in several phase I/II clinical trials using DNA electrotransfer^4,5^. Our knowledge on electrotransfer has been obtained both through theoretical and empirical approaches, but, while it is already used for wide industrial and medical applications, there are still major uncertainties and sustained controversies about the molecular and supramolecular effects of electrical pulses allowing the plasma membrane crossing by bioactive agents^2,6–10^.

It is more and more evident that electrotransfer mechanisms are dependent on the size of the penetrating object. The concept of hydrophilic pore formation induced by electric pulses in the membrane bilayer (“electroporation” phenomena) is probably relevant to the penetration of small molecules (up to a few nanometers), although we lack precise information on the nature and size of these pores^11–13^. Studies trying to visualize the reality of electropores, predicted by molecular dynamics computations, are scarce and controversial^14,15^. The concept of pore is clearly insufficient when dealing with penetration of large molecules, especially DNA, and a more complex course of events appears to have more relevance^16,17^. In this scenario, during the pulses, electropores appear in the plasma membrane, but more significantly, lipids are then oxidized in larger areas around generated pores^16^. The penetration of macromolecules seems to occur through these oxidized areas in a second step called “electropermeabilization”, which lasts much longer (up to several minutes) after electric pulse application, until the plasma membrane resealing. Several studies have actually shown that DNA electrotransfer was a multistep process consisting of (1) cell membrane electropermeabilization, (2) DNA electrophoretic drift towards electropermeabilized sites, (3) DNA-membrane interactions, (4) translocation of the DNA across the membrane few minutes after the pulses^18,19^. This model is supported by studying in vitro the roles of cell electropermeabilization and DNA electrophoretic drift with, respectively, high voltage (HV) and low voltage (LV) pulses^20–24^, and also by earlier observations made on gene transfer in vivo^25–27^. The real nature of the translocation step is still unknown yet. Some authors have noticed that electric fields could stimulate the uptake of various macromolecules by electro-endocytosis^28–30^, including plasmid DNA^31^. But, while many publications seem to confirm the involvement of endocytosis in the translocation step^32–40^, contradictory reports evidenced DNA entry by a non-endocytic translocation through the permeabilized membrane^18,19,26,41–43^. Several theoretical models, more or less based on experimental results, were proposed to explain the “translocation step” of DNA electrotransfer, but no definitive reliable mechanism has been described so far.

Interestingly, there are several reports where electrotransfer is used for biomolecules distinct from nucleic acids, like proteins (β-galactosidase, bovine serum albumin or full antibody), or dextran^28–30,44^. However, to our knowledge, there is no report on the use of electrotransfer as a tool to induce or facilitate the intracellular penetration of viral particles. The first aim of the present study was to determine whether it was possible to extend the application of electrotransfer to nano-objects of such large size, by testing different electric pulse types. As a model system, we chose to use as a penetrating agent a recombinant adenovirus (rAd) expressing a reporter gene, and as a target a murine cell line, L929, which is found weakly transduced by human adenovirus type 5 (Ad5)^45^. Human Ad5-based vectors have been widely studied in molecular biology and used in preclinical research and in gene therapy protocols. The fight against COVID-19 pandemic has hastened its deployment as a recombinant virus-vectored vaccine in the regular population. Ad5 does not replicate efficiently in most rodent cells, however, murine as well as human tissues can be transduced with Ad5-based vectors which efficiently express their carried transgene^46^. Ad5 initiates its cell entry by interaction between the fiber knob and the Coxsackie virus and Adenovirus Receptor (CAR), an adhesion molecule of the human host^47^, with its murine homolog, mCAR, found in the mouse^48^. Once bound at the cell surface, Ad5 is taken up into clathrin-coated pits by receptor-mediated endocytosis^49,50^. When CAR gene is weakly or not expressed, especially in some cancer cells^51^, Ad5 can bind cells by using other proteins of the host (in particular integrins α_3_β_1_ and α_v_β_5_) but with a lower efficacy^52^. Therefore, virus particles cannot be considered as classical non-permeant molecules for which cell electropulsation facilitates their direct penetration into the cytosol^11^. Furthermore, in contrast to naked DNA, once it has crossed the plasma membrane, Ad5 avoids the multiple impediments that may hinder its gene expression. In addition, there is no topological variation of structure that can influence virus entry and expression unlike plasmid DNA^53^. Therefore, in a given cell type, we can directly estimate the virus penetration into cells by measuring the expression of the reporter gene, inserted into the viral genome.

In the event of positive results, our next objective was to explore the mechanisms of viral penetration induced by electric pulses. The expected benefits of this work are of two kinds: (1) in terms of basic knowledge, to get more insights about electric pulse impact on the interactions between targeted cells and large penetrating agents; (2) in terms of practical applications, to open new ways for virus-based gene therapies applied for example to target tissues with weak or no expression of virus receptors (particularly in tumor or muscle); or to promote efficient entry of mutated (detargeted) recombinant viruses into cells to be treated for therapeutic purposes.

## Materials and methods

### Cell culture

L-929 (ATCC-CCL-1), a subclone of murine strain L fibroblastic cell line and 293 (ATCC-CRL-1573), a human epithelial cell line isolated from human embryonic kidney and containing left end of Adenovirus 5 DNA (LGC Standards, Molsheim, France), were grown in DMEM medium + GlutaMAX with 1% non-essential amino acids (Gibco, ThermoFisher Scientific, Watham MA, USA) and 10% FBS (Invitrogen, ThermoFisher Scientific). CHO-hCAR was kindly provided by J.M. Bergelson; this epithelial-like cell line derives from a subclone of Chinese Hamster Ovary cell line, CHO-K1 (ATCC-CCL-61), stably transformed with a plasmid coding human CAR^47^. It was grown in F-12 HAM (Gibco) with 10% FBS. G418 sulfate (geneticin, Gibco) was added at a final concentration of 250 µg/ml to maintain selective pressure on transformed cells. FBS had been previously heated (30 min, 56 °C) to inactivate complement and filtered on a 0.22 µm filter to remove protein aggregates. Before further processing, 60–70%-confluent cells were detached from dishes with TrypLE Express solution (Life Technologies, Courtabeuf, France), centrifuged (235×*g*, 10 min, 4 °C), counted, and suspended in S-MEM (Gibco), which was previously incubated in a CO_2_-incubator (Heracell 240i, ThermoFischer Scientific), to maintain its pH around 7.

### Adenovirus preparation, titration and cell infection

A rAd, Ad-CMV-eGFP, expressing the enhanced Green Fluorescent Protein (GFP) under the control of the cytomegalovirus (CMV) early promoter was constructed and the virus stocks were purified using standard procedures as described^54^. Briefly, the rAd was amplified on 293 cells that were harvested around 35 h after infection, disrupted by 5 cycles of freezing/thawing and cell debris were removed by centrifugation (2000×*g*, 10 min, 4 °C). The supernatant containing the crude viral solution was purified by two successive CsCl equilibrium density gradients (90,000×g, 2 h and 100,000×g, 18 h, respectively) and CsCl was removed by gel filtration on a Sephadex G-25 PD-10 column (Cytiva Europe, Velizy-Villacoublay, France). Virus aliquots were stored at − 80 °C in the conservation medium (10 mM Tris/HCl pH 7.5, 10% glycerol). Viral solution was assayed on 293 cell monolayers for plaque forming units (pfu) and titrated by HPLC^55^ to estimate viral particles (vp) number. Vp/pfu ratio around 20 was obtained, in agreement with published values^56^. Potential alterations that would be caused by electric pulses on rAd were also tested by end-point dilution assays on 293 cells, before and after electric pulse application on viral suspension: no appreciable variations were found by comparing both results.

The NanoSight LM10 nanoparticle characterization system (Malvern Instruments Ltd, Malvern WR, UK), equipped with a blue laser (405 nm) illumination, was used for adenovirus characterization (particle size and distribution) after stock batch thawing. A mean capsid diameter of 110 nm was found in accordance with the literature. The small aggregates (of 2–3 particles) represented less than 10% of the viral particles. Adenovirus surface charge was also measured, using a Zeta potentiometer (Nanobrook 90PLUS PALS, Brookhaven Instruments Corporation, Holtsville NY, USA) equipped with a 35 mW red diode laser, 640 nm wavelength. Ad particles were diluted in PBS (pH 7) and Zeta potential was measured at 25 °C. A mean value of − 20.95 mV (n = 5, SEM = 1.79) was found, indicating a low charge of adenovirus at physiological pH. The rAd was diluted in cold S-MEM without FBS (viral suspension) and added to cells, held on ice, either before or after electric treatment, at the multiplicity of infection (MOI in vp/cell) indicated. Uninfected controls contained similar dilutions of the virus conservation medium alone.

### Electric pulse delivery and treatment protocols

Microsecond high voltage electric pulse train (HV: 8 × 100 µs, 1 Hz, 130 kVm^−1^) and millisecond low voltage electric pulse train (LV: 10 × 20 ms, 10 Hz, 10 kVm^−1^, unless otherwise specified) were delivered by a Cliniporator device (IGEA, Carpi, Italy). A 10 dB-attenuator was connected inline after the Cliniporator to deliver LV pulses below 12 kVm^−1^. Experiments incorporating bipolar pulses or unipolar pulses with alternate polarities were conducted using an Electro cell B10 electropulser (βTech, Saint-Orens de Gameville, France). LV pulses delivered had the same parameters as those used for unipolar pulses, except inversion of polarity after 10 ms during the 20 ms-bipolar pulses, and after each pulse for pulses with alternate polarities. Schematics of electric pulses are shown in Supplementary Fig. S1.

For pulse delivery, 5 × 10^6^ cells/ml in suspension in 50 µl of S-MEM without FBS, held 15 min on ice, were put in a standard cuvette with 1 mm-spaced plate aluminum electrodes (Cell Projects, Arrietsham, UK). Non-pulsed controls were held in a cuvette in the same conditions but without pulse delivery. Except where indicated, cells were maintained on ice between 20 and 40 min after treatment; then, they were collected and diluted 200 times in complete medium at room temperature, distributed in 6-well plates in two technical replicates per experimental point, and incubated at 37 °C.

To determine the percentage of electropermeabilized cells, the non-permeant YO-PRO-1 (trimethyl-[3-[4-[(*Z*)-(3-methyl-1,3-benzoxazol-2-ylidene)methyl]quinolin-1-ium-1-yl]propyl]azanium; diiodide) dye (LifeTechnologies, Carlsbad CA, USA) was added (1 µM), just before the application of pulses and cells, kept on ice, were examined 40 min later by flow cytometry analysis.

### Gene expression analysis

The GFP gene expression was analyzed according to two parameters: the acquisition of GFP expression by target cells—in other words the conversion rate to GFP-positive (GFP^+^) cells, in % of GFP^+^ cells, and the GFP gene expression intensity—median of cell fluorescence intensity, in Relative Fluorescent Units (RFU). Cells were analyzed 24 to 72 h after the rAd-mediated gene transfer (AMGT) by flow cytometry. Treated cells (in duplicates) from 6-well plates were washed with 500 µl of PBS, trypsinized with 500 µl of TrypLE™ Express for 5 min at 37 °C, and wells were finally washed with 500 µl of complete medium. Cells were suspended and immediately analyzed by the flow cytometer (BD Accuri C6, Becton Dickinson, Franklin Lakes NJ, USA). GFP fluorescence was determined by setting the excitation at 488 nm, the emission at 530/30 nm with a BP filter, and recording 10,000 events per sample. GFP fluorescence parameters were analyzed using the BD Accuri CFlow Plus software. The gating strategy for flow cytometry is described in Supplementary Fig. S2.

The GFP cell conversion with time was observed using a real-time live cell imaging apparatus (IncuCyte Zoom, Essen Bioscience, Ann Arbor MI, USA) to determine GFP expression kinetics from 2 up to 35 h after the AMGT. Cells were plated immediately after the electric pulse delivery into 6-well plates (in technical duplicates) at densities which were adjusted to keep subconfluence during the experiment. The dishes were left in incubator at 37 °C during 2 h to allow cells to settle at the bottom of the wells before introducing them into the imaging apparatus. The mean percentage of GFP-expressing cells was calculated from 18 (2 × 9) fields distributed into duplicated dishes and imaged every 3 h (see representative pictures taken by the imaging apparatus camera in Supplementary Fig. S3). Cell eccentricity (equals to 0 for round cells and tends toward 1 for elliptical fully adherent cells) was also calculated at each time point, by the IncuCyte Zoom software.

### Aggregate observation, characterization, quantification, and experimental protocols

Aliquots (100 µl) of various solutions with a neutral pH (S-MEM culture medium; Phosphate Buffered Saline (PBS): 137 mM NaCl, 2.7 mM KCl, 10 mM Na_2_HPO_4_, 1.8 mM KH_2_PO_4_; Hepes-NaCl: 25 mM 2-[4-(2-hydroxyethyl)piperazin-1-yl]ethane-1-sulfonic acid, 130 mM NaCl) were pulsed with low voltage pulses (20 × LV), or left unpulsed. After mixing with 4 ml of sterile water in 6-cm dishes, they were observed by optical microscopy (Evos XL Core, ThermoFischer Scientific), using a phase contrast 20-objective. The protocol used for the determination of aggregate elemental composition and quantification by Inductively Coupled Plasma-Atomic Emission Spectroscopy (ICP-AES) is described in Supplementary Table S1. Pulsed medium (S-MEM) or pulsed viral suspension (S-MEM + rAd) were obtained after low voltage pulse delivery (10 × LV) either to the medium or to the viral suspension, in absence of cells. For dose–response study, different fractions of pulsed viral suspension (from 0% up to 100%) were mixed with complementary fractions of unpulsed viral suspension. The protocol used for the analysis of GFP L929 cell conversion as a function of chemical aluminum hydroxide quantities added to the viral suspension is described in Supplementary Fig. S4.

### Measurement of cell viability

The percentage of GFP^+^ cells presented in the figures corresponds to the percentage of living cells expressing GFP. The number of living cells for each experimental value was determined by flow cytometry, using the BD Accuri CFlow Plus software. More precisely, cells grown as adherent cultures were washed with PBS to remove all the floating dead cells. Living cells were then detached with TrypLE Express and immediately analyzed by the flow cytometry. Classical forward and side scatter gating was used to identify live cells, while removing debris, cell fragments, pyknotic cells, etc (see the gating strategy in Supplementary Fig. S2). The Accuri C6 allows the precise determination of the sample volume (using a microprocessor-controlled peristaltic pump) in addition to the fluorescence intensity and cell number for any gated population, allowing thus the absolute cell counts of the live cells. Viability of L929 or CHO-hCAR cells at different time intervals after treatment was expressed as a percentage versus the number of living cells counted in the untreated samples (set at 100% viability).

Long term viability was also appreciated using a colony formation assay. Briefly, after treatment, cells (5 × 10^6^ cells/ml in 50 µl), left 40 min in the cuvette at ambient temperature, were serially diluted in complete medium to obtain about 200 cell colonies per 4 cm-well for the non-treated control, and duplicate wells were filled with 3 ml of the ultimate cell dilution. After a 6-day incubation at 37 °C in a CO_2_-cell incubator, culture medium was aspirated and wells were gently washed with 2 ml of PBS. Cell colonies were fixed and stained for 10 min with Crystal Violet solution (0.5 g in 80 ml distilled water/20 ml methanol), then washed with distilled water. After complete drying, colonies were counted in each well in duplicate.

### Statistical analysis

**Figure 1:**
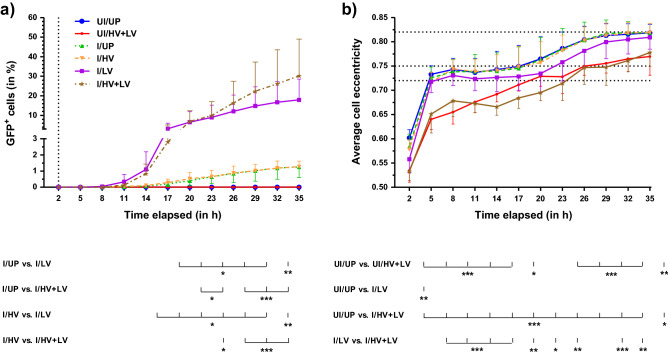
Real-time monitoring of GFP conversion of L929 cells after adenovirus infection and delivery of various types of electric pulses. (**a**) Percentage of GFP-converted cells as a function of elapsed time (in h), for each type of electric pulse. GFP^+^ cells percentage resulted from the ratio of fluorescent adherent cell number to total adherent cell number assessed by image analysis using the IncuCyte Zoom software. For optimal presentation of the initial stage of GFP expression, Y-axis is broken to indicate the change of scale above 3%. (**b**) Average cell eccentricity variations (from 0 up to 1) as a function of elapsed time (in h), calculated using the IncuCyte Zoom software. These variations reflect adherent cell recovery several hours after treatment. Plateau values of eccentricity (at 0.72, 0.75 and 0.82) are indicated by horizontal dotted lines. Treatment procedures for L929 cells were as indicated in the “Materials and methods” section, with the MOI set at 4 × 10^4^ vp/cell. Image acquisition started 2 h after the treatment (vertical dotted line on the graph (**a**) and was repeated every 3 h for 35 h. Abbreviations and curve symbols: *UI* uninfected, *I* infected, *UP* unpulsed, *LV/HV/HV* + *LV* application of low, high, high plus low voltage pulses, respectively; UI/UP (blue filled asterisk), UI/HV + LV (ref filled circle), I/UP (green filled triangle), I/HV (yellow filled inverted triangle), I/LV (violet filled square), I/HV + LV (gold filled star). Data are presented as means ± SD (with n = 3 for each condition). Error bars are indicated either up or down, depending on experimental points, for optimal visualization of the curves. Statistical significances: *p < 0.05, **p < 0.01 and ***p < 0.001 stand for comparisons of GFP^+^ cells in (**a**) and average cell eccentricity in (**b**), at each time interval and between different experimental conditions indicated below both graphs (two-way ANOVA for repeated measures with Bonferroni’s post-test).

Data representative of several independent experiments (n = 3 to 6) are presented as means ± SD, with experimental points in duplicate for each independent experiment (except where indicated). For experiments including comparison between various conditions, one- or two-way ANOVA, (or a two-way ANOVA for repeated measures for Fig. 1) was applied. A Bonferroni’s multiple comparison test or, for experiments comparing all columns versus one control column, a Dunnett’s test was used as a post-hoc test. When data failed the variance homogeneity test, a non-parametric analysis was done instead, one- or two-way ANOVA on ranks with subsequent Tukey post-hoc test. *p < 0.05 was considered statistically significant. For experiments with measure of fluorescence intensity, data were previously log_10_-converted before application of a comparison test. All graphs and statistical analyses were realized with GraphPad Prism 5 and SigmaStat 4.0 softwares, respectively.

## Results

### Effect of different types of electric pulses on adenovirus-mediated gene transfer

Adenovirus-mediated gene transfer (AMGT) was assessed by infection of L929 with a rAd encoding the GFP gene. Acquisition of GFP expression by target cells was measured by flow cytometry at 48 h post-infection (Table 1) or by continuous real-time imaging during 35 h (Fig. 1). The percentage of GFP^+^ cells and the median of fluorescence intensity (only for flow cytometry analysis) were used as indices of AMGT, itself proportional to the cell penetration of viral particles.

**Table 1:** Comparison of GFP gene expression and survival of L929 and CHO-hCAR cells following adenovirus infection and delivery of various types of electric pulse.

Cell line	Measured variable	Experimental conditions							
		UI/UP	I/UP	UI/HV	I/HV	UI/LV	I/LV	UI/HV + LV	I/HV + LV
(**a**) L929	GFP^+^ cells (living cells %)	0.03 ± 0.01	47.00 ± 8.34	0.04 ± 0.02	58.82 ± 9.34	0.03 ± 0.01	98.25 ± 2.44** ^#^	0.01 ± 0.01	98.42 ± 1.43** ^#^
	Fluo. int. (in log_10_ RFU)	3.78 ± 0.13	4.52 ± 0.03	3.86 ± 0.12	4.58 ± 0.05	3.83 ± 0.47	5.47 ± 0.32* ^#^	4.15 ± 0.71	5.96 ± 0.33*** ^#^
	Cell survival (% of UI/UP)	100.00	96.13 ± 5.91	100.88 ± 7.99	90.52 ± 10.40	41.44 ± 12.60°°°	49.70 ± 17.89°°°	6.38 ± 4.08°°°	8.39 ± 7.48°°°
(**b**) CHO-hCAR	GFP^+^ cells (living cells %)	0.02 ± 0.01	82.67 ± 3.62	0.02 ± 0.01	82.36 ± 2.98	0.03 ± 0.03	75.78 ± 2.56	0.01 ± 0.00	79.18 ± 2.01
	Fluo. int. (in log_10_ RFU)	3.58 ± 0.36	5.37 ± 0.05	3.62 ± 0.08	5.41 ± 0.05	3.88 ± 0.33	5.32 ± 0.07	3.55 ± 0.24	5.49 ± 0.05^**$**^
	Cell survival (% of UI/UP)	100.00	97.09 ± 4.98	106.43 ± 5.98	95.24 ± 4.12	44.97 ± 2.04°°°	43.25 ± 3.43°°°	6.21 ± 1.30°°°	15.87 ± 4.98°°°

(**a**) L929 and (**b**) CHO-hCAR were treated with the same procedures, except for the viral MOI (10^4^ vp/cell and 50 vp/cell, respectively). GFP^+^ cells (in % of living cells), median of fluorescence intensity (in log_10_ RFU) and cell survival (in % of UI/UP cells) were scored by flow cytometry, 48 h after the treatment.

*UI* uninfected, *I* infected, *UP* unpulsed, *LV/HV/HV* + *LV* application of low, high, high plus low voltage pulses, respectively, *fluo. int*. fluorescence intensity.

Data are presented as means ± SD (with n = 6 for L929 and n = 3 for CHO-hCAR cells). Statistical significances: *p < 0.05, **p < 0.01, and ***p < 0.001 stand for comparisons of GFP^+^ cells and fluorescence intensity versus I/UP condition; ^#^*p* < 0.05 and ^$^*p* < 0.05 for the same comparisons versus I/HV or I/LV condition (one-way ANOVA on ranks with Tukey post-test or one-way ANOVA with Bonferroni’s post-test for L929 and CHO-hCAR, respectively); °°°*p* < 0.001 for comparison of cell survival versus UI/UP condition (one-way ANOVA with Dunnett’s post-test).

First, the effects of several types of electric pulses on AMGT were tested for two cell lines: L929, weakly transduced by Ad5, and CHO-hCAR, which exhibits, on the contrary, an excellent rate of adenovirus infection. In the absence of electric pulses (Table 1, I/UP condition), the GFP conversion rate was only around 47% and the fluorescence intensity 3.30 × 10^4^ RFU, using a MOI of 10^4^ vp/cell for L929 cells, in contrast with about 83% and 2.34 × 10^5^ RFU, respectively, using only 50 vp/cell for CHO-hCAR cells. This reflects the low rate of viral penetration inside L929 cells, indicating a low expression of specific viral receptors. In subsequent experiments on L929 cells, the viral MOI was set at 0.5–1 × 10^4^ vp/cell as the optimal magnitude to allow the detection of potential positive or negative effects of electric pulses on rAd cell penetration. The effects of several types of electric pulses on the AMGT and on the cell survival were tested for infected L929 (Table 1a) or CHO-hCAR (Table 1b) cells. Regarding L929, low voltage (LV) pulses alone had a significant increasing effect, both in terms of GFP conversion and cell fluorescence intensity. A minor influence of HV pulses delivered prior to LV ones was mainly observed for the fluorescence intensity, since 98% of cells were already GFP^+^ with LV pulses alone (Table 1a). With the electric parameters used, short term survival was much more affected by LV treatment (around 50% survivors) and even more for HV + LV treatment (8%), than by HV treatment alone (90%). Rather, in CHO-hCAR cells, although cell survival was affected by electric treatments in similar proportions than in L929 cells, the AMGT was not modified by electric pulse application (Table 1b). This is likely because they already exhibit an excellent rate of adenovirus infection at baseline, associated to their high levels of hCAR transgenic expression.

When using a real-time monitoring of GFP cell conversion for 35 h following electric pulses, we confirmed that LV pulses significantly enhanced rAd penetration, while HV pulses alone had no effect unless they were combined with LV pulses (Fig. 1a). It should be noted that the detection threshold of the fluorescence emitted by the GFP was much higher (roughly 50–60 times more) for the real-time imaging device camera (see Supplementary Fig. S3) than for the flow cytometer detector, explaining the quantitative differences between the results of these two types of experiments. Moreover, the real-time monitoring of the average cell eccentricity showed that LV, and even more HV + LV treatments, in line with cell survival results obtained with the flow cytometry experiments (see Table 1), also transiently impaired the capacity of infected cells to attach to dishes, compared to HV pulse treatment or unpulsed cells (Fig. 1b). Indeed, the curves tend to reach a maximal eccentricity value between 0.72 and 0.82, when untreated cells (UI/UP) achieved full adherence. The delay required to cross the eccentricity threshold of 0.75 was about 17 h for unpulsed controls (± infection) and the HV condition, about 22 h for the LV condition, and 26–29 h for HV + LV-treated cells (± infection). In addition, we observed a delayed onset of measurable GFP conversions for I/HV + LV versus I/LV conditions (see Fig. 1a). Therefore, the curve divergence observed 20 h after treatment, could be explained by a disturbed and delayed cell proliferation for I/HV + LV-compared to I/LV-treated cells, resulting in lower dilutions of GFP fluorescence in daughter cells and a higher percentage of GFP^+^ *detected* at delayed time of experiment. This effect also explains the higher fluorescence intensity per cell observed for I/HV + LV- compared to LV-treated cells, 48 h after electric pulse application in both cell lines (see column I/HV + LV in Table 1). Altogether, these data support the idea that HV pulses are not efficient to enhance AMGT in L929 cells and that LV pulses are required to obtain a significant increase in GFP cell conversion.

### Optimization of LV pulses and viral MOI for AMGT enhancement in L929 cells

**Figure 2:**
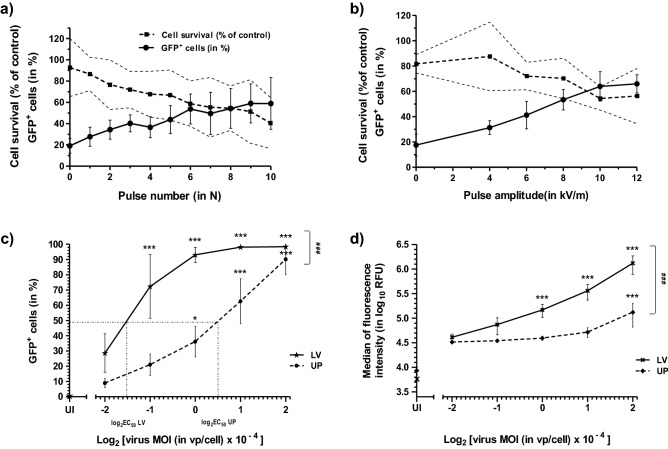
Influence of various experimental parameters on GFP gene expression and survival of L929 cells subjected to adenovirus infection and LV pulse application. GFP^+^ cells (in %) and cell survival (in % of UI/UP cells) as a function of: (**a**) the number (in N) of LV pulses delivered, (**b**) the amplitude of LV pulses (in kVm^−1^). Infected L929 cells (MOI = 10^4^ vp/cell) were subjected to (**a**) increasing numbers of LV pulses at 12 kVm^−1^, or (**b**) ten LV pulses of increasing voltages up to 12 kVm^−1^, 48 h prior to flow cytometry. Abbreviations and curve symbols for (**a**,**b**) graphs: GFP^+^ cell % (black filled circle), with error bars, cell survival (black filled square), with error (thin dotted lines). Data are presented as means ± SD (with n (**a**) = 5 and n (**b**) = 3). (**c**) GFP^+^ cells (in %) and (**d**) median of fluorescence intensity (in log_10_ RFU), as a function of the viral MOI used for infection (half dilutions from 4 × 10^4^ to 0.25 × 10^4^ vp/cell were converted into a log_2_ for graphic considerations). L929 cells were mixed with rAd or with the vehicle alone (UI condition) and left unpulsed (UP condition) or subjected to LV pulses and analyzed by flow cytometry, 27 h post treatment. In (**c**), half maximal effective virus concentrations (EC_50_) for LV and UP conditions have been indicated. Abbreviations and curve symbols for (**c,d**) graphs: *UI* uninfected, *UP* unpulsed, *LV* low voltage pulses; GFP^+^ cell % when UP (black filled circle) or after LV (black filled star), and median of fluorescence intensity when UP (black filled diamond) or after LV (black filled cross). Data are presented as means ± SD (with n = 4 for (**c,d**) ). Statistical significances for (**c,d**): *p < 0.05 and ***p < 0.001 stand for intra-curve comparisons of GFP^+^ cells and fluorescence intensity versus the lowest MOI condition; ^**###**^*p* < 0.001 for UP and LV curve comparison (two-way ANOVA with Bonferroni’s post-test for (**c**), two-way ANOVA on ranks with Tukey post-test for (**d**).

GFP conversion of L929 cells was investigated as a function of LV pulse number and intensity. For a given MOI, the rate of conversion was enhanced in the same proportion as the number of pulses and their intensity in a range of 4 kVm^−1^ up to 10 kVm^−1^ (Fig. 2a,b, respectively). However, the increase of GFP cell conversion was achieved at the cost of a decline in cell survival which was, for example, below 50% for 10 pulses at 12 kVm^−1^ (Fig. 2a). Cell survival was also affected by the raise of pulse amplitude, at least above a threshold of about 4 kVm^−1^ (Fig. 2b). Since the rate of GFP conversion did not further increase beyond 10 kVm^−1^, we set up this amplitude value for all subsequent experiments.

GFP conversion and fluorescence intensity were then investigated as a function of the viral MOI (Fig. 2). The GFP conversion rate was increasing in the same proportion as the viral MOI up to a threshold of 60–70%, for both UP and LV pulse conditions (Fig. 2c). Then, the curves flattened to progressively reach a plateau where up to 98% of L929 cells expressed GFP. However, comparing these two dose–response curves from the calculation of their half maximal effective virus concentrations (EC_50_): EC_50_LV = 0.35 × 10^4^ vp/cell and EC_50_UP = 1.41 × 10^4^ vp/cell, the application of LV pulses resulted in about a four-fold viral MOI reduction corresponding to a boost of the cell transduction efficacy. The fluorescence intensity in relation to the viral MOI (Fig. 2d) rather showed segmental positive linear regression for UP and LV pulse conditions, with a breakpoint at a MOI value of 2 × 10^4^ and 0.5 × 10^4^, respectively, otherwise corresponding to 60–70% of GFP cell conversion in both conditions (see Fig. 2c). At the ultimate MOI used, fluorescence intensity was 0.13 × 10^6^ RFU for the UP cells and about tenfold more for LV-pulsed cells (Fig. 2d). Thus, when nearly all the cells have been GFP-converted, the LV pulse treatment also increased fluorescence intensity several fold.

### Investigations on plasma membrane modifications related to LV pulses

**Figure 3:**
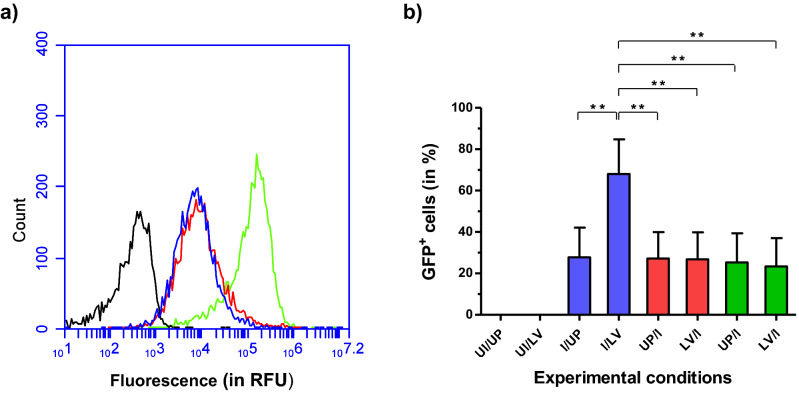
Contribution of electropermeabilization and electrophoretic effect in the GFP conversion enhancement induced by LV pulse application in infected L929 cells. (**a**) Flow cytometry detection of intracellular YO-PRO-1 to assess electropermeabilization of L929 cells subjected to HV versus LV pulse application. L929 cells were exposed to YO-PRO-1, immediately subjected to HV or LV pulses (amplitude = 12 kVm^−1^) and, 40 min later, to flow cytometry analysis (excitation: 488 nm, emission: 530/30 nm). The graph represents cell distribution (count) according to fluorescence (in RFU) by the YO-PRO-1 dye (logarithmic scale). Cells not exposed to YO-PRO-1 (black line, auto-fluorescence), cells exposed to YO-PRO-1 without application of electric pulses (blue line) or subjected to LV or HV pulses (red and green line, respectively). (**b**) GFP^+^ cells (in %) as a function of the order and time interval between LV pulse application and rAd infection. L929 cells were infected with rAd at MOI = 10^4^ vp/cell, 5 min before (blue columns) or 1 min (red columns) to 5 min (green columns) after LV pulse delivery. The GFP^+^ cells were scored by flow cytometry 48 h after the treatment. *UI* uninfected, *I* infected, *UP* unpulsed, *LV* application of low voltage pulses. Data are presented as bar chart, with means ± SD (n = 5). Statistical significance for (**b**): **p < 0.01 stands for comparisons between experimental conditions as indicated by the brackets on the graph (one-way ANOVA on ranks with Tukey post-test).

Initially, we assumed a role for plasma membrane electropermeabilization to explain this result. To investigate this mechanism, we have used the non-permeant dye YO-PRO-1 during cell electroporation using either HV or LV pulses. As shown by flow cytometry analysis in Fig. 3a, cell penetration of YO-PRO-1 was induced by HV but not by LV pulses used in our experiments, evidencing that an electroporation process was not involved in the enhanced penetration of rAd in L929 cells. To consider the potential role of an electrophoretic effect exercised particularly by long-lasting pulses, we made a comparison of the effects of LV pulses delivered to L929 cells, either prior or after exposure to viral particles. We found that AMGT was enhanced only when both cells and rAd were subjected to LV pulses (Fig. 3b). This result suggested that an electrophoretic effect of LV pulses on viral particles could contribute to the observed effect, but also indicated that direct modifications of the plasma membrane by electric pulses were not critical for the AMGT increase.

### Observation, characterization, and quantification of aggregated particulate material

**Figure 4:**
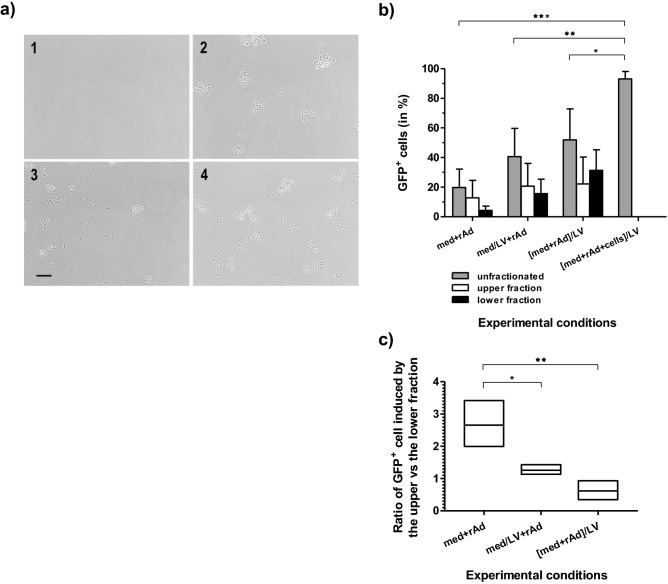
Observation of aggregated particles after LV pulse application and their role in the enhancement of GFP conversion in infected L929 cells. (**a**) Aggregated particle observation by optical microscopy (magnification: × 200), after application of twenty LV pulses on aliquots of three different solutions: (1) negative control (S-MEM unpulsed), (2) S-MEM pulsed, (3) PBS pulsed, (4) Hepes/NaCl pulsed. Size scale corresponding to the black bar is 80 µm. Photo editing was realized with Gimp 2.1 software. (**b**) Role of aggregated particles in the enhancement of GFP gene expression. L929 cells were infected with rAd (final MOI of 10^4^ vp/cell) using three types of inoculum: (1) viral suspension made in crude culture medium without any electric pulse (med + rAd); (2) viral suspension made using culture medium pulsed prior to virus mixing (med/LV + rAd); (3) viral suspension made in crude culture medium but pulsed after virus mixing ([med + rAd]/LV); for the last experimental condition, LV pulses were directly applied on cells, already mixed with viral suspension ([med + rAd + cells]/LV). In addition, for the first three experimental conditions, the viral suspension was either left 10 min at 4 °C (unfractionated inoculum indicated in grey) or subjected to a short centrifugation (300×*g*, 10 min, 4 °C) in Eppendorf tubes, yielding a upper (40 µl) and a lower (10 µl) fractions (indicated in white and black, respectively). Each fraction was then separately added to L929 target cells and GFP cell conversion was assessed by flow cytometry 24 h post treatment. *UP* unpulsed, *LV* application of low voltage pulses, *med* medium, *rAd* recombinant adenovirus. Data are presented as bar chart, with means ± SD (n = 3, except ultimate condition where n = 4). Statistical significances: *p < 0.05, **p < 0.01, and ***p < 0.001 stand for comparisons between experimental conditions (without fractionation) indicated by the brackets on the graph (one-way ANOVA with Bonferroni’s post-test). (**c**) The ratios of GFP cell conversion induced by the upper versus the lower fraction are presented for the first three experimental conditions as vertical boxes (minimum to maximum value), with central line indicating the mean and Y-axis indicating the ratio values. Statistical significances: *p < 0.05 and **p < 0.01 stand for the ratio comparisons indicated by the brackets on the graph (one-way ANOVA with Bonferroni's post-test).

Since there was no evidence that the enhancement of AMGT by LV pulses was mainly related to plasma membrane modifications, we explored the influence of LV pulses on the culture medium itself. A first step was made by simple observation under a phase-contrast microscope of an aliquot of culture medium subjected to LV pulses or not. We detected a particulate material which formed precipitable aggregates specifically in the pulsed medium (Fig. 4a, pictures 1–2). To confirm that the presence of these aggregates was due to the application of electric pulses and was independent of the solution used, we reproduced the same experiment with two other solutions, simpler than the initial culture medium (Fig. 4a, pictures 3–4). In each case, we observed the formation of qualitatively similar aggregates after application of LV pulses.

Next, in order to characterize the nature of these aggregates indisputably, the material released in each of the three solutions (S-MEM, PBS and Hepes/NaCl), pulsed or not pulsed (as negative controls), was subjected to a highly sensitive analysis: the Inductively Coupled Plasma-Atomic Emission Spectroscopy (ICP-AES). This technique allows both the elemental composition and quantification of the samples (see Supplementary Table S1). This experiment has established that the aggregates were made of aluminum (hydrogen and oxygen cannot be detected by this method). Aluminum (Al) was found in the different solutions only after application of pulses to the different solutions and the quantity produced were independent of their composition but depended on the number of LV pulses, thus, this metal emanates perforce from the energized electrodes (according to the Faraday’s second Law). At pH 7, Al is mainly found as aluminum hydroxide (Al(OH)_**3**_), insoluble in water, and aqueous Al^3+^ ions, resulting from anodic oxidation during application of pulse, are quickly transformed in Al(OH)_**3**_ when the pH > 4. To confirm this fact in our experimental conditions, we added a control in the ICP-AES experiment where the pellet, obtained after pulse application to the PBS solution and centrifugation, was washed with distilled water to eliminate the dissolved salts (including potential soluble Al^3+^ ions). This control showed the same Al content formed (around 0.32 µg for one LV pulse) than the unwashed samples, indicating that Al(OH)_3_ is the unique source of aluminum in neutral physiologic solutions (see Supplementary Table S1).

### Effect of the aluminum hydroxide aggregates on AMGT increase

To establish that these metallic particles were involved in the AMGT enhancement, L929 cells were infected using four experimental conditions: (1) viral suspension unpulsed, (2) made with medium pulsed only (formation of aggregates before mixing with rAd), (3) pulsed (formation of aggregates in the presence of rAd), (4) viral suspension mixed with cells and pulsed together (formation of aggregates in the presence of rAd and cells). Moreover, in the three first conditions, viral suspension was finally added to target cells either unfractionated or fractionated by low-speed centrifugation in upper and lower fractions, the latter being enriched in aluminum aggregates of various sizes (Fig. 4b). Strikingly, delivery of LV pulses to the viral suspension, or even to the plain medium before virus addition to cells, was sufficient to enhance AMGT compared with unpulsed condition (Fig. 4b, unfractionated inocula). Moreover, in each condition, the benefit was greater for the lower fraction than for the upper one, indicating a specific contribution of aluminum aggregates to viral particle entry (Fig. 4b, fractionated inocula). This interpretation was confirmed by the data obtained when medium and virus were pulsed together: not only with the unfractionated viral suspension AMGT was greater than with previous conditions, but after fractionation, the relative contribution of the lower fraction was also strongly increased, suggesting a role for co-aggregation of virus and aluminum particles resulting from concomitant exposure to electric pulses (see Fig. 4c, ratio of the upper fraction to the lower one). An ultimate and large increase was observed when LV pulses were also applied on cells mixed with the viral suspension (Fig. 4b).

**Figure 5:**
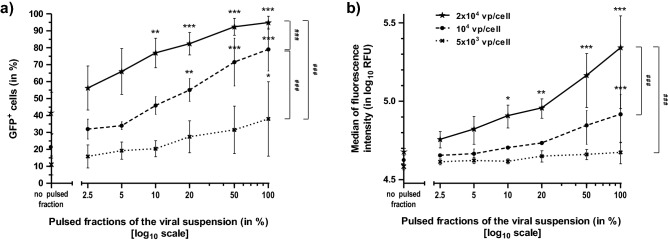
Analysis of GFP gene expression in L929 cells as a function of viral MOI and percentage of the viral suspension treated with LV pulses. S-MEM medium was mixed with rAd to obtain viral suspensions adjusted at three different MOI in vp/cell: 5 × 10^3^ (black filled cross), 10^4^ (black filled circle), and 2 × 10^4^ (black filled star), when added to L929 cells. Pulsed fractions of the viral suspension (from 2.5 to 100%) were indicated on the X-axis, in log_10_ scale for the sake of clarity. They were added to L929 cells that were analyzed by flow cytometry 48 h post treatment. (**a**) GFP^+^ cells (in %), or (**b**) median of fluorescence intensity (in log_10_ RFU) are presented. By comparison, the median level of L929 cell fluorescence in the absence of GFP expression (auto-fluorescence of uninfected cells treated with 100% of pulsed medium) was 3.77 ± 2.72 log_10_ RFU. Data are presented as means ± SD (n = 3). Statistical significances for (**a,b**): *p < 0.05, **p < 0.01, and ***p < 0.001 stand for intra-curve comparisons of GFP^+^ cells and fluorescence intensity versus the “no pulsed fraction” condition; ^**###**^*p* < 0.001 for comparisons between curves representing different virus MOI (two-way ANOVA with Bonferroni’s post-test).

Additional steps were taken to confirm the role of Al(OH)_**3**_ particles in the AMGT enhancement. First, we have made an experiment comparing the effects of unipolar LV pulses to those of bipolar LV or unipolar LV pulses with alternated polarity (Table 2). The enhancement of viral penetration was greatly reduced with these two latter pulse types, known to produce less metallic particles than unipolar LV pulses of the same intensity and duration^57^. Furthermore, to test a dose-dependent effect of Al(OH)_3_ aggregates, we realized infections made with mixtures of pulsed and unpulsed viral suspensions. For each virus MOI, the rate of GFP conversion was proportional to the pulsed fraction size (from 0 to 100%). Each curve reached a plateau whose value was dependent on the MOI, i.e. on the quantity of available viral particles (Fig. 5a). However, additional increase in fluorescence intensity was detected even beyond the point where the rate of GFP conversion reached a plateau of almost 100%, for the highest viral MOI used (Fig. 5b). This indicated that Al(OH)_**3**_ particles acted both on GFP conversion rate and on cell fluorescence intensity.

**Table 2:** Comparison of GFP gene expression and survival of L929 cells after adenovirus infection and delivery of various LV pulse types.

Cell line	Measured variable	Experimental conditions							
		UI/UP	I/UP	UI/unipolar	I/unipolar LV	UI/bipolar LV	I/bipolar LV	UI/altern. LV	I/altern. LV
L929	GFP^+^ cells (living cells %)	0.03 ± 0.01	40.99 ± 2.91	0.02 ± 0.01	96.56 ± 0.40***	0.01 ± 0.00	51.06 ± 4.40* ^###^	0.01 ± 0.01	49.59 ± 2.26* ^###^
	Fluo. int. (in log_10_ RFU)	3.79 ± 0.15	4.61 ± 0.01	4.02 ± 0.16	5.31 ± 0.02***	3.80 ± 0.12	4.66 ± 0.02^###^	3.70 ± 0.19	4.65 ± 0.01^###^
	Cell survival (% of UI/UP)	100.00	107.80 ± 17.31	57.53 ± 20.51	50.04 ± 17.42	82.66 ± 31.30	91.46 ± 9.85	80.3 ± 22.30	84.81 ± 17.84

L929 cells were infected (MOI = 10^4^ vp/cell) and subjected to ten LV pulses either unipolar, bipolar, or with alternate polarities, respectively. GFP^+^ cells (in % of living cells), median of fluorescence intensity (in log_10_ RFU) and cell survival (in % of UI/UP cells) were scored by flow cytometry analysis 24 h ± 30 min after the treatment.

*UI* uninfected, *I* infected, *UP* unpulsed, *LV* application of low voltage pulses, *fluo. int.* fluorescence intensity, *altern.* alternate.

Data are presented as means ± SD (n = 3). Statistical significances: *p < 0.05 and ***p < 0.001 stand for comparisons of GFP^+^ cells and fluorescence intensity versus I/UP condition; ^**###**^*p* < 0.001 for the same comparisons versus I/unipolar LV condition (one-way ANOVA with Bonferroni’s post-test).

**Figure 6:**
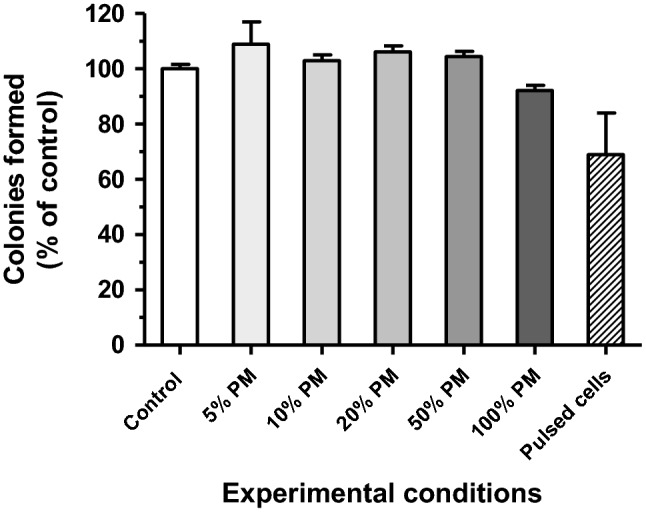
Long term viability of non-infected L929 cells treated with increasing fractions of pulsed medium or direct LV pulse application. Culture medium was subjected to LV pulses in the absence of cells. Then, L929 cells (5 × 10^6^ cells/ml) were incubated for 40 min in a mix of crude and pulsed medium with increasing proportions of the latter, through six experimental conditions (0% to 100% PM). They were then diluted in complete DMEM medium and plated into duplicated 4 cm-wells (technical replicates). In one additional experimental condition, cells were directly subjected to LV pulses before the colony formation assay (rightmost column). Colonies were counted in each well after 5 days of culture and counts were expressed in percentages of the control condition (0% PM). *PM* pulsed medium, *LV* low voltage. Data are presented as bar chart, with means ± range of technical replicates.

To verify if aluminum hydroxide from another source (than that from electrolysis reactions) could also have a detectable effect, we realized an additional experiment where we mixed the rAd with Al(OH)_**3**_ from a chemical supplier and analyzed the effect on GFP conversion of L929 cells (see Supplementary Fig. S4). We used increasing amounts of Al(OH)_3_, with a range (0.08 to 39 µg) including the quantity observed in the culture medium after one LV pulse train (10 × 20 ms): around 9 µg (see Table S1). Consistently, though lower, we observed an enhanced adenovirus entry in L929 cells with similar quantities of aluminum hydroxide from both origin (compare Fig. 4b with Fig. S4) and the dose–effect ranges were also comparable (compare Fig. 5a with Fig. S4). Finally, the experiment depicted in Fig. 6 showed that long-term viability of cells treated with various fractions of pulsed medium was much better preserved than that of cells directly exposed to LV pulses.

## Discussion

We investigated whether electric pulses could facilitate cell penetration of large biological objects, as viruses, and chose to evaluate the electrotransfer potential of a rAd encoding GFP by measuring the AMGT efficacy. First, we have indeed observed a substantial increase in AMGT resulting from the application of LV millisecond- (but not HV microsecond-) electric pulses, unlike standard protocols used for DNA electrotransfer. Second, we tried to understand the underlying mechanisms of this increase. Initially, our hypothesis was that the penetration of viral particles was facilitated by a combination of electropermeabilization and electrophoresis. But the AMGT increase occurred in the LV-treated L929 cells in the absence of their electropermeabilization as demonstrated by the absence of YO-PRO-1 uptake. In contrast, above given thresholds, the LV-increased GFP expression was proportional to the number of pulses applied (total duration of the electric field application) and to their voltage. These data suggested a role for an electrophoresis-dependent uptake of viral particles by attracting them towards the charged cells, surprising knowing that the charge carried by the adenovirus capsid is very low. However, an electrophoretic effect hypothesis was also supported by the fact that pulse delivery must be done in presence of the virus to be efficient. It became obvious that the mechanism(s) of AMGT increase were not directly due to an effect of electric pulse on the cells, since LV pulse application on L929 cells, several minutes before rAd infection, could not show any impact on virus entry such as an electro-endocytosis process.

To go further, we analyzed whether the LV pulses could have an effect on the virus, namely on the viral suspension. Strikingly, AMGT was enhanced when viral particles were subjected to electric pulses before they come into contact with the cells, indicating that the main effect of electric pulses was rather a modification of the penetrating agent. Since delivery of the pulses on culture medium, prior to addition of the viral particles, was sufficient to get the AMGT enhancement, we suspected the contribution of a facilitating material released from the electrodes, energized during electric pulse delivery. This was confirmed by the detection, characterization, and quantification of Al in aggregates, reproducibly found in several solutions subjected to millisecond pulses.

Several reports have studied the emissions of metallic ions, mainly as undesirable modifications of the experimental conditions and detrimental effects on target cells^57–62^. In particular, one group has observed that electric pulses induced significant precipitation of biological macromolecules (DNA, RNA and proteins) in electroporation using millisecond pulses^59^. The authors have shown that aluminum electrodes caused greater precipitation than stainless steel electrodes. The suggested mechanism was that positively charged metal ions, immediately during their generation with the pulses, interact with the negatively charged residues of nucleic acids or proteins, neutralizing the repulsive forces between macromolecules and facilitating the aggregate formation. It should be noted, that this mechanism is commonly used at an industrial scale for the wastewater treatment under the term of electrocoagulation. This might lead to possible artifacts and false interpretations of gene electrotransfer efficiency. In our experiments, the formation of large aggregates of aluminum hydroxide (visible under optical microscopy as observed in Fig. 4a) is probably due to (1) the use of electric millisecond pulses that induced the production of Al^3+^ ions in higher amounts, (2) the neutral pH of the solution used, promoting their rapid and total transformation in precipitable Al(OH)_3_, (3) their concentration in the vicinity of electrodes and the electrophoretic force exercised by repeated millisecond pulses, favoring their aggregation. Overall, the release of aluminum ions in the medium modifying the experimental conditions became an attractive hypothesis. Next, we have shown qualitatively that the AMGT increase detected was mainly due to the presence of the aggregating Al(OH)_3_ particles formed during millisecond pulses in the conducting medium (see Fig. 4b,c), and quantitatively, since the effect was directly proportional to the particulate material quantity added to cells (see the dose–effect curve in Fig. 5).

Friedrich and collaborators have concluded that aluminum cuvettes could be used for experiments, provided that the pulse duration time was in the range of 10–100 µs and amplitude below 400 kVm^−1^ (Al^3+^ concentration not exceeding 60 µM in that case). Under these conditions, plasmid electrotransfer in several cell lines (including L929) seemed not disturbed by aluminum particles in the medium^63^. In our conditions, long term survival of uninfected L929 cells directly treated with LV pulses can be under 60% of non-treated cells (Fig. 6). In contrast, we observed a long-term survival above 90% when these cells were exposed to a full dose of Al(OH)_3_ particles, formed during LV pulse application to the crude medium (Fig. 6). Thus, the toxicity seems given mainly by direct exposition of cells to LV pulses rather than through an external effect of Al(OH)_3_ particles or of other electrolytic species generated by these pulses. The pH modification due to an exposure to electric pulses has been studied: this phenomenon is more particularly important with aluminum electrodes and use of millisecond pulses^64^. Furthermore, pH variations in the electrolytic chamber can exceed 1 to 2 pH units in average and pH fronts are the principal cause of tissue damage near the electrodes^64,65^. The unbuffered change of pH, improved by each pulse, might be one of the factors triggering the cell death. This cumulative harmful impact on survival rate is clearly shown in Fig. 2a. In the case of HV + LV pulses, the penetration of electrolytic products inside cells, permeabilized by the HV pulses, might also explain the very low survival rate observed (see Table 1). Several groups have proposed means to reduce these electrolytic contaminations, among others by replacing unipolar rectangular pulses by bipolar pulses^58,63,64,66^. So, as a complementary approach, we compared the effects obtained when using bipolar or alternate pulses instead of monopolar pulses (Table 2). Since Al^3+^ cations are produced around electrodes, we have also speculated that using alternate pulses at 10 Hz frequency would have a similar effect than real bipolar pulses.

Ultimately, we have some clues to understand the intimate mechanism(s) of AMGT improvement. A lower AMGT increase was observed with Al(OH)_3_ from chemical origin (compare Fig. 4b and Supplementary Fig. S4), indicating that qualitative factors matter (different sizes of particles or aggregation capacity?). The GFP conversion rate increased proportionately with the quantity of Al(OH)_3_ particles in the medium and reached a plateau, proportional to the virus concentration (Fig. 5a). This would mean that (large) aggregates of Al(OH)_3_ particles, combined with virions, probably promote more numerous cell encounters (considering the effect of aggregate size, density or/and global charge) than “free” virus, resulting in more cells expressing at least one copy of GFP gene. Besides, we have shown that more virions were associated to Al(OH)_3_ aggregates when these latter were generated from the viral suspension (Fig. 4b,c). This was probably due to Al^3+^ ions reactivity with neighboring viral particles during their generation with the pulses (interaction with the negatively charged residues of viral capsid proteins), associated to their electrophoretic drift towards the virions, leading to precipitable Al(OH)_3_-virion aggregates. Then, the cell fluorescence intensity actually increased with increasing amounts of Al(OH)_3_ aggregates and virions, only when nearly all cells had been GFP-converted, i.e. when they had at least one viral particle expressing GFP (Fig. 5b). This could mean that more virions, associated to Al(OH)_3_ aggregates, should penetrate together (and/or enter more frequently) per cell with LV pulse delivery, explaining the differences observed between curves, at the highest viral MOI used (Fig. 2d). All together, these results imply that the mechanism of cell penetration is more efficient for the Al(OH)_3_ aggregate-associated virus than for the free virus in L929 cells. Finally, if we compare the latest bar of Fig. 4b with those of the same figure, and the data of Fig. 2c,d with those of Fig. 5a,b, respectively, we observe that AMGT was always improved when cells were present during pulse application. This difference can be interpreted by the role played again by the electrophoretic effect of LV pulses, pushing metallic/virus aggregates towards cell surface.

## Conclusion

In the cell line and electric conditions tested here, we came to the conclusion that the main contribution of the electric pulses to enhanced adenovirus penetration was not a modification of the target cell but rather a modification of the penetrating agent. We have demonstrated that the increased entry of adenovirus particles in L929 cells after application of LV pulses, essentially resulted from virus association with large aluminum aggregates formed during the electric impulses from aluminum plate electrodes. When virus particles, and even more when both cells and virions are present during the pulses, the electrophoretic effect could also play an important role. It is likely, even if not proven here, that the virus/metallic aggregate complex enters cells through a (or several) endocytosis process(es), including macropinocytosis, considering the large sizes of some aggregates observed. These results should hence be generalized to several types of human cells that are naturally weakly or not transduced by adenovirus vectors, including primary cancer cells, differentiated endothelial or epithelial cells, and mesenchymal or hematopoietic stem cells. Adenovirus 5 receptor (CAR) is frequently deficient in these cells, resulting in low penetration of viral particles. Many attempts to improve adenovirus entry in vitro and in vivo focused on virus complexation with polycations (cationic lipids or polymers), facilitating its binding to negatively-charged cell membrane. But these substances have frequently shown cytotoxic effects. Recently a functionalized magnesium phyllosilicate (aminoclay) as has been shown to enhance AMGT through nanobiohybrid complex formation with Ad particles, aggregating them and making their surface charge highly positive, thereby providing an additional cellular entry mechanism by caveolae-dependent endocytosis^67^. Besides, increasing evidence indicates that some viruses naturally spread also in virion groups such as aggregates or in secreted lipid vesicles. These structures increase the MOI independently to the viral population density and contribute to the maintenance of viral genetic diversity. In their natural hosts, this could have implications for, *inter alia*, virus transmission, genetic complementation between mutants and innate immunity evasion^68^. What can we learn about plasmid DNA (pDNA) electrotransfer mechanisms from this study? The diameter of the linear form of pDNA is in order of 2–3 nm, while that of the supercoiled pDNA of 4.7 kbp is one order of magnitude larger^53^. These diameters, unlike that of Ad5, remain largely compatible with a pDNA translocation into cells following an “electropermeabilization” process. Ultimately, comparative investigations of electrotransfer for various nano-objects (including other viruses) must be continued for a deeper understanding of internalization processes and safety of future therapeutic use.

## Supplementary Information

Supplementary Information.
