# Supplementary material for: Aluminum particles generated during millisecond electric pulse application enhance adenovirus-mediated gene transfer in L929 cells
Source: Sci Rep. 2021 Sep 6;11:17725. doi: 10.1038/s41598-021-96781-y (PMC8421418; doi:10.1038/s41598-021-96781-y)
Supplement: Supplementary file 1 — Supplementary Information. [file 41598_2021_96781_MOESM1_ESM.docx]

**Aluminum particles generated during millisecond electric pulse application enhance adenovirus-mediated gene transfer in L929 cells**

**Authors**

Angela Tesse^2^, Franck M André^1^, and Thierry Ragot^1🖂^

**Supplementary Material**


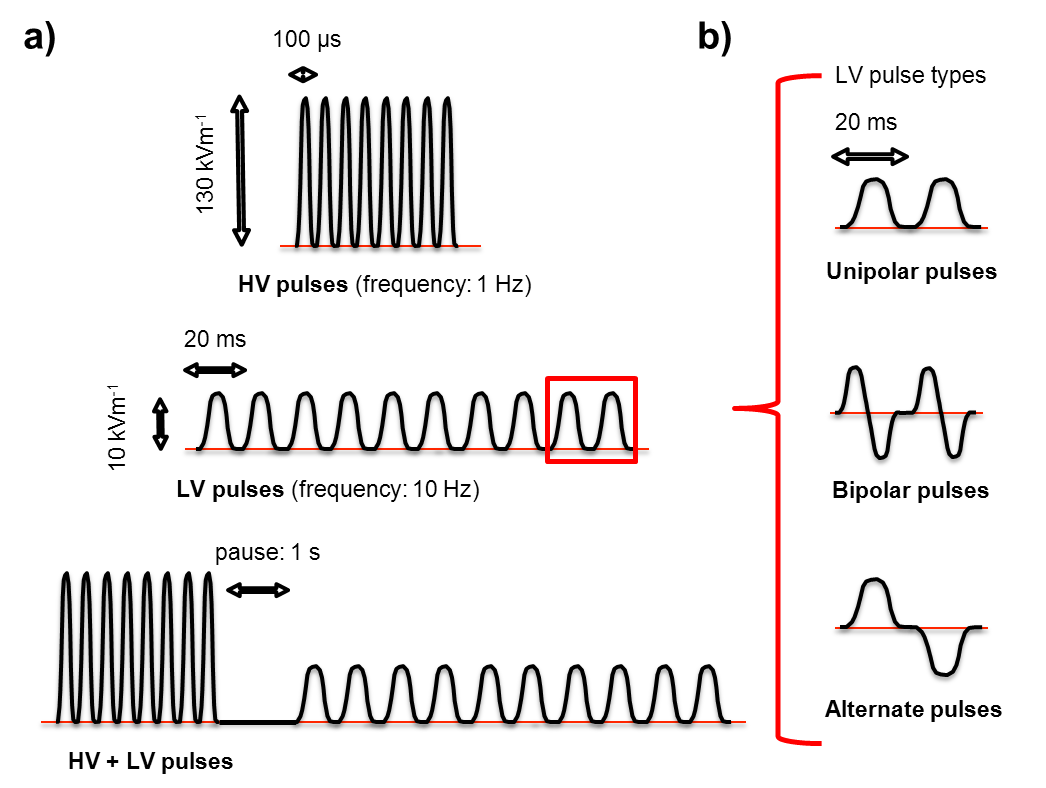


**Supplementary Figure S1. Schematics of the various electric pulses used in the experimental protocols. a)** Various pulse trains applied on infected L929 or CHO-hCAR cells. Microsecond high voltage pulses (HV), millisecond low voltage pulses (LV) and a combination of HV + LV pulses (see Table 1). **b)** Different types of LV pulses applied on infected L929 and CHO-hCAR cells: unipolar, bipolar and alternate (polarity) pulses (see Table 2).


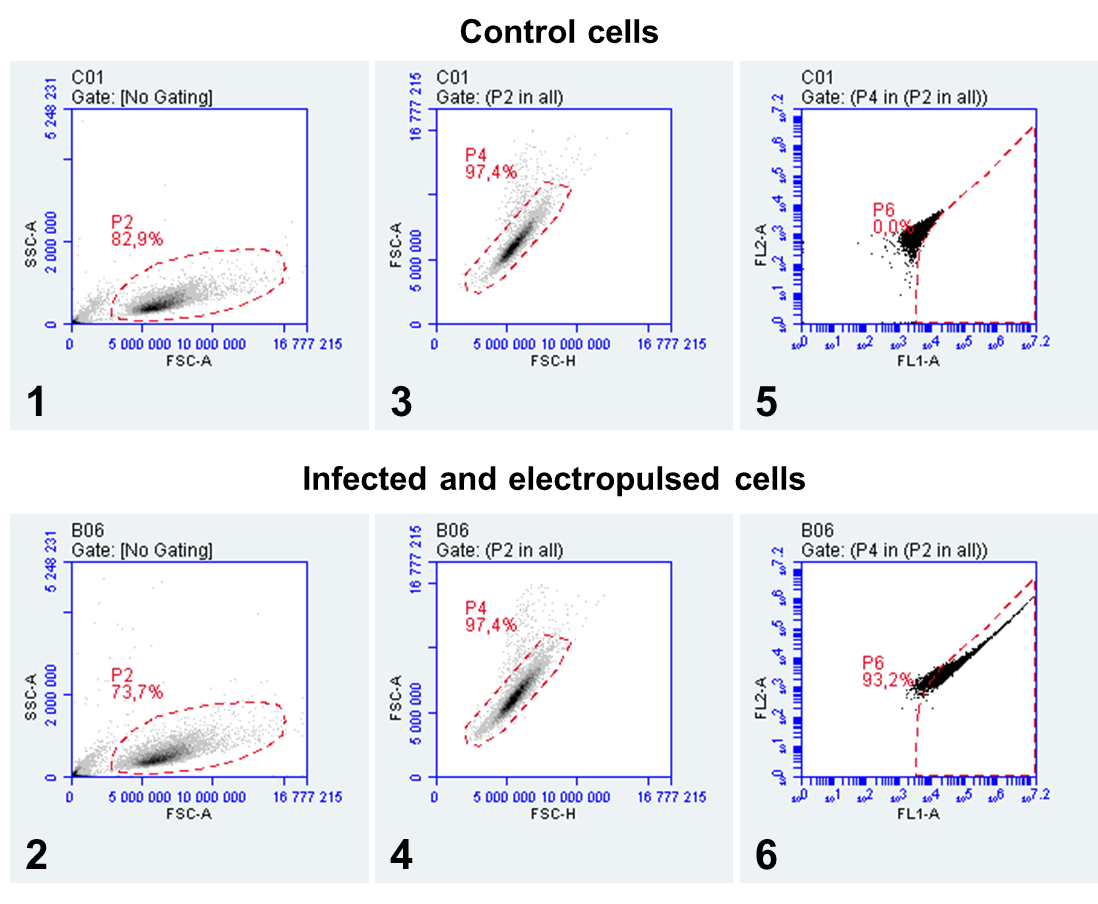


**Supplementary Figure S2. Gating strategy for flow cytometry.** Representative cytometry graphs of control (higher panel) and infected and electropulsed cells (lower panel) showing the gating strategy to determine the L929 viability and percentage of GFP^+^ cells after adenovirus infection and pulse application. **1-2**: forward scatter area (FSC-A) vs. side scatter area (SSC-A) plot was used to gate the cell population and remove the debris. We used large gating (P2) to consider morphologic variations of cells and eliminated events with low FSC-A and SSC-A, and those with low FSC-A and high SSC-A to ensure the gating of the majority of viable cells. **3-4**: forward scatter height (FSC-H) vs. FSC-A plot was used to select single cells by choosing a diagonal gating (P4). **5-6**: FITC channel (FLT1) vs. PE channel (FLT2) plot was used to gate GFP^+^ cells in order to eliminate any fluorescence component due to an auto-fluorescence increase. The gating was based on the uninfected control cells to ensure a threshold of false positivity below 0.1 % in the region of interest chosen (P6).


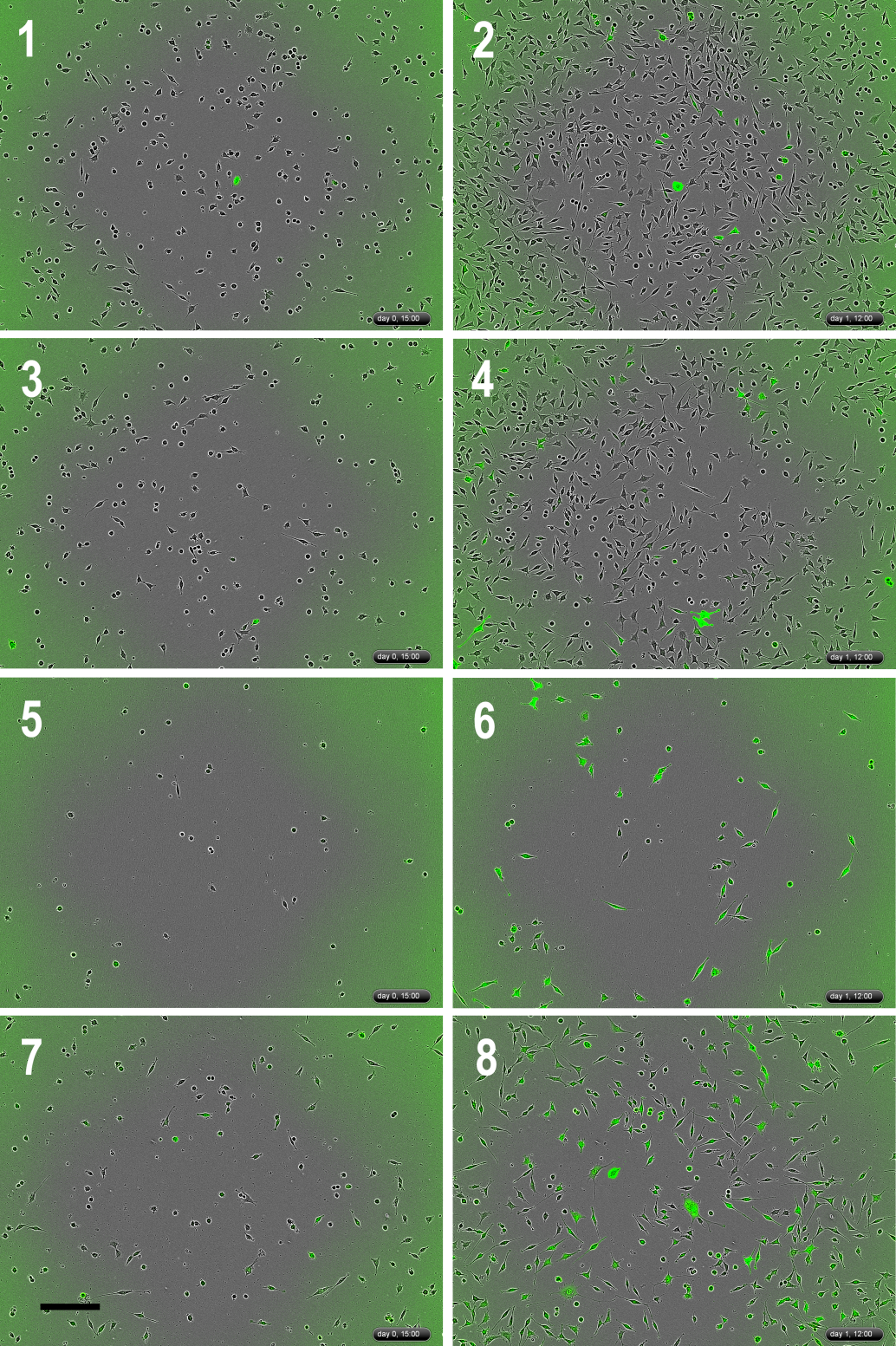


**Supplementary Figure S3. Microphotographies of L929 cells during real-time monitoring of GFP cell conversion.** Representative images, taken by the real-time imaging apparatus camera (20 x-objective) in phase contrast and green fluorescence, were chosen for two time intervals after the beginning of experiment (left panel: 15 h, right panel: 35 h). Different experimental conditions were shown, respectively from top to bottom, **1-2**: infected unpulsed cells (I/UP); **3-4**: infected high voltage-pulsed cells (I/HV); **5-6**: infected high voltage- + low voltage-pulsed cells (HV+LV); **7-8**: infected low voltage-pulsed cells (I/LV). Size scale corresponding to the black bar is 80 µm.

|  |  |  | |  |
| --- | --- | --- | --- | --- |
| **Experimental conditions** | | | | **Results** |
| **Medium** | **LV pulse train number** | | **Treatment** | **Total Al mass (mean +/- SD) in µg** |
|  |  | |  |  |
| S-MEM | 0 | | Speedvac concentration | -0.64 ± 0,39 |
| S-MEM | 20 | | Speedvac concentration | 62.87 ± 0,96 |
| PBS | 0 | | Speedvac concentration | -0.60 ± 0,38 |
| PBS | 20 | | Speedvac concentration | 66.30 ± 1,25 |
| PBS | 40 | | Centrifugation + pellet washing | 133.52 ± 0,57 |
| Hepes-NaCl | 0 | | Speedvac concentration | -0.66 ± 0,35 |
| Hepes-NaCl | 20 | | Speedvac concentration | 66.89 ± 0,77 |
|  |  | |  |  |
|  |  | |  |  |

**Supplementary Table S1. Determination of aggregate elemental composition and quantification of aluminum by Inductively Coupled Plasma–Atomic Emission Spectroscopy (ICP-AES).** Various solutions with neutral pH (S-MEM culture medium; Phosphate Buffered Saline (PBS): 137 mM NaCl, 2.7 mM KCl, 10 mM Na_2_HPO_4_, 1.8 mM KH_2_PO_4_; Hepes-NaCl: 25 mM 2-[4-(2-hydroxyethyl)piperazin-1-yl]ethane-1-sulfonic acid, 130 mM NaCl) were pulsed with low voltage pulses (LV: 10 x 20 ms, 10 Hz, 10 kVm^-1^), or left unpulsed in cuvettes (0 pulse). Number of LV pulse train (10 x 20 ms) is indicated (pause between two successive pulse trains: 1 min). Then, pulsed or unpulsed solutions were submitted either to a 20-fold concentration in a Speedvac (AES 1010 Speedvac concentrator, Savant ThermoFischer, Watham MA, USA) or to a centrifugation in Eppendorf tubes (9200×g, 20 min, 4°C), pellet washing with sterile distilled water, followed by a second centrifugation in the same conditions. Qualitative elemental composition and precise determination of Al amount were determined using ICP–AES analysis with a iCAP 6300 radial ICP emission spectrometer (ThermoFischer Scientific) at the Laboratoire de Planétologie et Géodynamique (LPG) - UMR 6112 CNRS, Nantes University. The samples were analyzed to determine their elemental composition. Two multi-element solutions (SQS01, SQS02, Chem-Lab, Zeldegem, Belgium) were used to determine the different elements present in the solutions. Since aluminum is the unique element exclusively observed in the pulsed solutions, a mono-element solution of Al (1000 ppm, Chem-Lab) was used to determine precisely the amount found in each sample. For all samples, 1 ml of HNO_3_ 68% was added and they were ultrasonicated 10 min and left for 1 h before dilution to 20 g with ultrapure water. For each sample, five technical replicates were analyzed in the spectrometer and the final result is calculated from the mean of six Al emission lines (λ in nm = 236.7, 237.3, 308.2, 309.2, 394.4, and 396.1). In the table, the total Al mass by sample is given in µg ± SD (n=6). Unpulsed samples did not contain Al since their values were comparable to those of the blanks introduced in the analysis and which value was subtracted from each sample value. The mass of Al contained in the pulsed sample treated by centrifugation and pellet washing is twice that of the other pulsed samples, in agreement with the number of LV pulses applied in each case (40 versus 20).

**Supplementary Figure S4. Analysis of GFP gene expression in L929 cells as a function of aluminum hydroxide quantities added to the viral suspension.** 20 µl of S‑MEM was mixed with rAd adjusted at a MOI of 10^4^ vp/cell, when finally added to cells. Increasing quantities of powdered Al(OH)_3_ (CAS 21645-51-2, Merck, Darmstadt, Germany), extensively suspended in sterile water, were added to the viral suspension held on ice as indicated on the X‑axis, and mixed again (but with gentle pipetting). The mixture was immediately added to 5x10^6^ L929 cells in a final volume of 50 µl, the suspension was mixed once by gentle pipetting and held 20 min on ice. Then, L929 cells were collected and diluted 200 folds in complete medium at room temperature, distributed in 6-wells plates in two technical replicates per experimental point, and incubated at 37 °C. Cells were analyzed by flow cytometry 48 h post treatment. GFP^+^ cells (in %) as a function of Al(OH)_3_ quantities (expressed in µg, with MW**_Al(OH)3_** = 78 gr.mol^‑1^) were represented as bar chart, with means ± range of technical replicates. Abbreviations: UI: uninfected, I: infected, Al: Al(OH)_3_.
